# An easy-to-operate web-based calculator for predicting the progression of chronic kidney disease

**DOI:** 10.1186/s12967-021-02942-y

**Published:** 2021-07-03

**Authors:** Qian Xu, Yunyun Wang, Yiqun Fang, Shanshan Feng, Cuiyun Chen, Yanxia Jiang

**Affiliations:** 1 Health Management Center, First Affiliated Hospital of Nanchang University, Nanchang, 330006 Jiangxi China; 2 Academic Affairs Office, First Affiliated Hospital of Nanchang University, Nanchang, 330006 Jiangxi China; 3 Department of Endocrinology and Metabolism, Jingdezhen First People’s Hospital, Jingdezhen, 333000 Jiangxi China; 4 Department of Endocrinology and Metabolism, First Affiliated Hospital of Nanchang University, 17 Yongwai, Nanchang, 330006 Jiangxi People’s Republic of China

**Keywords:** Chronic kidney disease, End-stage renal disease, Area under the curve, Prognostic factor, Progression-free survival

## Abstract

**Background:**

This study aimed to establish and validate an easy-to-operate novel scoring system based on simple and readily available clinical indices for predicting the progression of chronic kidney disease (CKD).

**Methods:**

We retrospectively evaluated 1045 eligible CKD patients from a publicly available database. Factors included in the model were determined by univariate and multiple Cox proportional hazard analyses based on the training set.

**Results:**

Independent prognostic factors including etiology, hemoglobin level, creatinine level, proteinuria, and urinary protein/creatinine ratio were determined and contained in the model. The model showed good calibration and discrimination. The area under the curve (AUC) values generated to predict 1-, 2-, and 3-year progression-free survival in the training set were 0.947, 0.931, and 0.939, respectively. In the validation set, the model still revealed excellent calibration and discrimination, and the AUC values generated to predict 1-, 2-, and 3-year progression-free survival were 0.948, 0.933, and 0.915, respectively. In addition, decision curve analysis demonstrated that the model was clinically beneficial. Moreover, to visualize the prediction results, we established a web-based calculator (https://ncutool.shinyapps.io/CKDprogression/).

**Conclusion:**

An easy-to-operate model based on five relevant factors was developed and validated as a conventional tool to assist doctors with clinical decision-making and personalized treatment.

## Introduction

Chronic kidney disease (CKD), a common kidney disease with a progressive decline in renal function, is increasingly recognized as a global public health problem [1]. It causes more than half a million patients to develop end-stage renal disease (ESRD) every year, and over 700,000 deaths [2]. CKD is multifactorial and is defined as glomerular filtration rate (GFR) < 60 mL/min per 1.73 m^2^ or abnormalities in kidney structure or function present for more than 3 months [3, 4]. Diabetic nephropathy is the leading cause of CKD, accounting for approximately 40% of patients with non-dialysis-dependent CKD and ESRD [5]. Other pathological processes for CKD include chronic glomerulonephritis, ureteral obstruction, and renal fibrosis [6–8]. As effective therapeutic strategies for ESRD are currently limited, it is important to identify treatments to delay the progression of CKD to ESRD. Rapid CKD progression leads to irreversible pathological changes and may be associated with unfavorable outcomes. Once a patient has developed ESRD, renal replacement therapy is needed to maintain their daily activities. Therefore, there is an urgent need for reliable and accurate progression prediction models for CKD.

Many definitions of CKD progression have been used over the years, such as doubling of serum creatinine level, decrease in estimated GFR (eGFR) to < 15 mL/min per 1.73 m^2^, and development of ESRD [9, 10]. Currently, there are no clinically robust biomarkers to predict progressive CKD. Rather, clinicians rely on multiple longitudinal kidney measurements, such as the eGFR, proteinuria, and urinary protein/creatinine ratio (UPCR) to identify progression [11]. The shortcomings of these traditional biomarkers are well recognized, and a single index has limited predictive capacity for progressive CKD [12]. However, the use of complex and potentially expensive detection strategies may prevent at risk patients from benefiting from preventative interventions, especially in settings where renal replacement therapy is not readily available. The use of risk models is an attractive and likely cost-effective method for large-scale CKD risk stratification and would allow the identification of populations that would benefit the most from CKD detection. There have been several attempts to create a risk model for predicting the progression of CKD. However, the prediction accuracy of these models has not been tested through widespread application in clinical practice [13–16].

In the present study, we aimed to establish a model using Cox regression analysis based on commonly used and readily available clinical characteristics to predict disease progression in CKD patients. We performed univariate and multivariate analyses to screen for independent risk factors. The visualization model was constructed by nomogram and web-based calculator, and prediction performance was measured by discrimination, calibration, and clinical utility. This novel simple-to-use model might predict the prognosis of patients with CKD with high accuracy.

## Methods

### Ethics statement

The study was conducted in accordance with the ethical standards and the Declaration of Helsinki and according to national and international guidelines. It was approved by the authors' institutional review board (No. 883).

### Patients

This study used data from 1138 patients with CKD obtained via the Dryad Digital Repository (http://www.datadryad.org/), shared by Limori et al. [17]. According to Dryad's terms of service, researchers can use these data for secondary analysis without infringing on the author's rights. All eligible individuals who were not undergoing dialysis were diagnosed with stage G2–G5 CKD based on the Kidney Disease Improving Global Outcomes classification [18]. All participants were at least 20 years of age and visited nephrology centers for the first time between October 2010 and December 2011. Patients with malignancy that was diagnosed or treated within the previous 2 years, transplant recipients, and those with active gastrointestinal bleeding at enrollment were excluded. All eligible patients were randomly stratified into two groups in a 2:1 ratio (training set and validation set, respectively).

### Data collection

We performed a secondary analysis based on data from the above database. Fifteen probable prediction variables were selected, including gender, age, etiology (diabetes, nephrosclerosis, glomerulonephritis, and others), hemoglobin level, serum albumin level, creatinine level, eGFR, proteinuria, urinary occult blood, UPCR, hypertension, history of cardiovascular disease, diabetes, use of RAAS inhibitor, use of calcium channel blocker, and use of diuretics. Moreover, the vital status and follow-up time of each CKD case were extracted.

### Predictor selection and development of the prediction model

Depending on the training set, Cox proportion hazard regression models were used to screen potential prognostic factors and estimate their weights [19, 20]. Univariable Cox regression analysis was performed to explore the potential predictors [21]. The selected prognostic factors (p value below 0.05 in univariate analysis) were then included in a multivariate Cox regression analysis to obtain an integrated nomogram by a stepwise feature selection algorithm based on the AIC [22]. Moreover, to facilitate clinical application, we established a visualization tool by a web-based calculator.

### Validation of the prediction model

The performance of our model to predict survival was quantified using AUC values from the ROC analysis and the C-index. The performance of the novel model was also evaluated by examining calibration in training and validation sets. In addition, DCA was carried out to assess the clinical utility of the model. These tests were all performed in both the training and validation sets.

### Statistical analysis

Continuous variables following a normal distribution are presented as mean ± standard deviation and categorical variables are presented as percentages. Differences between the training and validation sets were analyzed using chi-square tests for the categorical variables and t-tests for the continuous variables. A p value < 0.05 was used as a cutoff for statistical significance. Statistical analysis was performed using SPSS software (version 24.0) and R software (version 3.6.2).

## Results

### Baseline characteristics

**Fig. 1:**
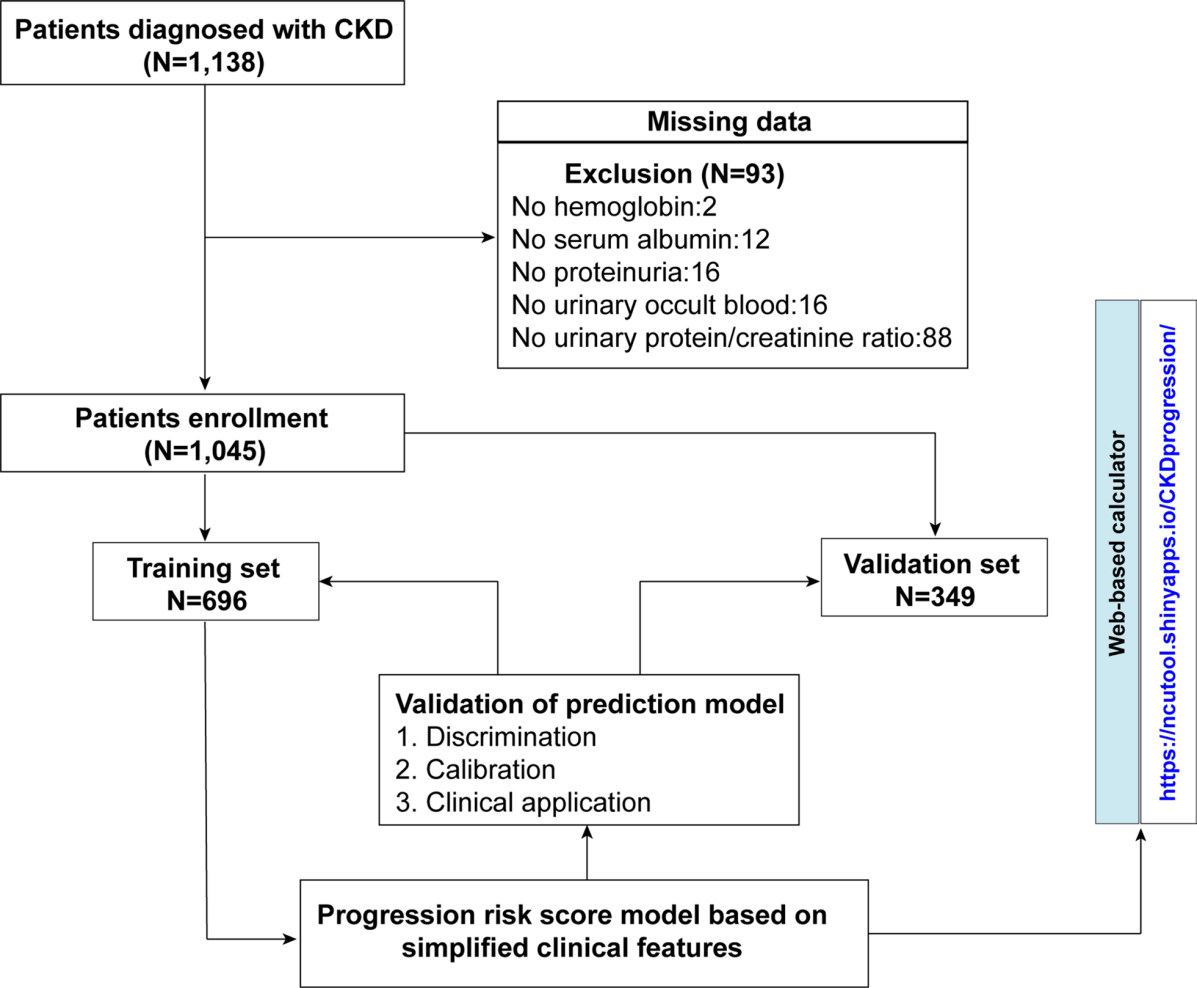
Flow diagram that shows the development and validation of the prediction model

Figure 1 shows a flow diagram of the selection process. After excluding 93 patients with missing data, a total of 1,045 patients was included in the analysis. Patients were randomly divided at a ratio of 6:3 into training (N = 696) and validation sets (N = 349). The demographics and clinical characteristics of the whole, training, and validation sets are presented in Table 1. In the whole cohort, 69.9% of the participants were male, and the mean age was 67.31 ± 13.6 years. Most patients had positive proteinuria and history of hypertension. Across the entire study population, 260 patients had disease progression (CKD progression defined as > 50% eGFR loss or initiation of dialysis).

**Table 1:** Baseline demographics and clinical characteristics of patients in training cohort and validation cohort

Variables	All patients (N = 1045)	Training set (N = 696)	Validation set (N = 349)	P-value
Gender, n (%)				0.966
Male	730 (69.9%)	487 (70.0%)	243 (69.6%)	
Female	315 (30.1%)	209 (30.0%)	106 (30.4%)	
Age, years	67.31 ± 13.60	66.70 ± 13.90	68.54 ± 12.90	0.039
Etiology, n (%)				0.759
Diabetic	271 (25.9%)	177 (25.4%)	94 (26.9%)	
Nephrosclerosis	411 (39.3%)	270 (38.8%)	141 (40.4%)	
Glomerulonephritis	197 (18.9%)	137 (19.7%)	60 (17.2%)	
Others	166 (15.9%)	112 (16.1%)	54 (15.5%)	
Hemoglobin, g/dL	11.97 ± 2.28	12.02 ± 2.29	11.87 ± 2.26	0.304
Serum albumin, g/dL	3.85 ± 0.63	3.87 ± 0.63	3.82 ± 0.64	0.211
Creatinine, g/dL	2.26 ± 1.72	2.25 ± 1.72	2.28 ± 1.71	0.825
eGFR, mL/min/1.73 m^2^	32.95 ± 18.82	33.15 ± 18.78	32.56 ± 18.90	0.632
Proteinuria, n (%)				0.919
Negative	381 (36.5%)	255 (36.6%)	126 (36.1%)	
Positive	664 (63.5%)	441 (63.4%)	223 (63.9%)	
Urinary occult blood, n (%)				0.361
Negative	689 (65.9%)	466 (67.0%)	223 (63.9%)	
Positive	356 (34.1%)	230 (33.0%)	126 (36.1%)	
UPCR, g/gCr	2.17 ± 3.24	2.04 ± 2.98	2.42 ± 3.69	0.073
Hypertension, n (%)				0.864
No	101 (9.7%)	66 (9.5%)	35 (10.0%)	
Yes	944 (90.3%)	630 (90.5%)	314 (90.0%)	
History of CVD, n (%)				0.183
No	765 (73.2%)	519 (74.6%)	246 (70.5%)	
Yes	280 (26.8%)	177 (25.4%)	103 (29.5%)	
Diabetes, n (%)				0.693
No	651 (62.3%)	437 (62.8%)	214 (61.3%)	
Yes	394 (37.7%)	259 (37.2%)	135 (38.7%)	
Use of RAAS inhibitor, n (%)				0.047
No	380 (36.4%)	238 (34.2%)	142 (40.7%)	
Yes	665 (63.6%)	458 (65.8%)	207 (59.3%)	
Use of calcium channel blocker, n (%)				0.616
No	547 (52.3%)	360 (51.7%)	187 (53.6%)	
Yes	498 (47.7%)	336 (48.3%)	162 (46.4%)	
Use of diuretics, n (%)				0.154
No	694 (66.4%)	473 (68.0%)	221 (63.3%)	
Yes	351 (33.6%)	223 (32.0%)	128 (36.7%)	
Vital status, n (%)				0.773
Alive	972 (93.0%)	649 (93.2%)	323 (92.6%)	
Deceased	73(7.0%)	47(6.8%)	26 (7.4%)	
CKD progression, n (%)				0.479
No	785 (75.1%)	528 (75.9%)	257 (73.6%)	
Yes	260 (24.9%)	168 (24.1%)	92 (26.4%)	

### Prognostic factors of CKD

Univariate Cox regression analysis showed that age, etiology, hemoglobin level, serum albumin level, creatinine level, eGFR, proteinuria, urinary occult blood, UPCR, hypertension, diabetes, use of renin–angiotensin–aldosterone-system (RAAS) inhibitor, use of calcium channel blocker, and use of diuretics were correlated with CKD progression. Multivariate Cox regression analysis identified etiology, hemoglobin level, creatinine level, proteinuria, and UPCR as independent prognostic factors of CKD patients (Table 2).

**Table 2:** Univariate and multivariable Cox hazards analysis of the training cohort

Variables	Univariate	*P*-value	Multivariate	*P*-value
	HR (95% CI)		HR (95% CI)	
Gender				
Male	Ref.	–	Ref.	–
Female	1.018 (0.734–1.412)	0.914		–
Age	0.989 (0.979–0.999)	**0.028**	0.998 (0.985–1.011)	0.720
Etiology				
Diabetic	Ref.		Ref.	–
Nephrosclerosis	0.147 (0.098–0.220)	**0.000**	0.540 (0.297–0.979)	**0.042**
Glomerulonephritis	0.230 (0.148–0.359)	**0.000**	0.437 (0.228–0.836)	**0.012**
Others	0.170 (0.097–0.299)	**0.000**	0.269 (0.118–0.618)	**0.002**
Hemoglobin	0.701 (0.655–0.751)	**0.000**	0.821 (0.749–0.900)	**0.000**
Serum albumin	0.267 (0.216–0.330)	**0.000**	0.869 (0.624–1.212)	0.409
Creatinine	1.429 (1.370–1.490)	**0.000**	1.314 (1.221–1.413)	**0.000**
Proteinuria				
Negative	Ref.		Ref.	
Positive	28.395 (10.53–76.571)	**0.000**	7.214 (2.547–20.436)	**0.000**
Urinary occult blood				
Negative	Ref.		Ref.	
Positive	2.156 (1.592–2.919)	**0.000**	1.096 (0.779–1.543)	0.597
UPCR	1.305 (1.264–1.348)	**0.000**	1.192 (1.126–1.261)	**0.000**
Hypertension				
No	Ref.		Ref.	
Yes	5.976 (1.908–18.719)	**0.002**	0.930 (0.271–3.197)	0.909
History of CVD				
No	Ref.	–	Ref.	–
Yes	1.366 (0.977–1.910)	0.068		–
Diabetes				
No	Ref.	–	Ref.	–
Yes	3.005 (2.205–4.096)	**0.000**	0.898 (0.527–1.528)	0.690
Use of RAAS inhibitor				
No	Ref.	**–**	Ref.	–
Yes	1.808 (1.259–2.595)	**0.001**	0.928 (0.627–1.374)	0.710
Use of calcium channel blocker				
No	Ref.	**−**	Ref.	–
Yes	2.024 (1.474–2.778)	**0.000**	1.298 (0.925–1.821)	0.132
Use of diuretics				
No	Ref.	**–**	Ref.	–
Yes	2.833 (2.092–3.836)	**0.000**	1.04 (0.741–1.461)	0.819

*P* < 0.05 is shown in bold

### Development of an individualized prediction model

**Fig. 2:**
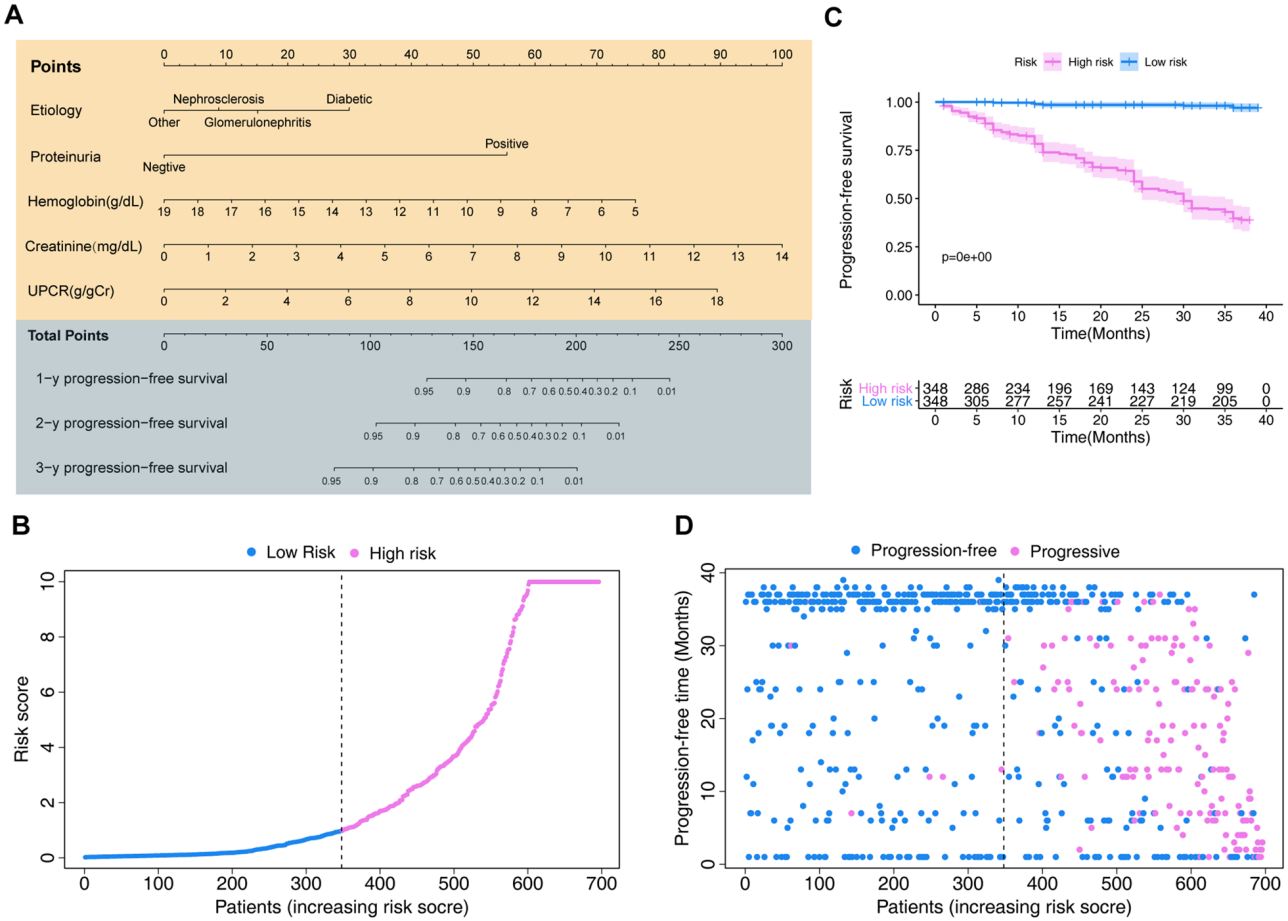
The model to predict the probability of progression in chronic kidney disease (CKD) patients from the training cohort. **A** The nomogram based on the five variables identified by the Cox hazards analysis. **B** Distribution of the risk scores calculated by the nomogram scoring system. **C** Progression-free survival curves stratified by the low- and high-score groups. **D** Patient distribution in the low- and high-score groups based on progression status

Based on Akaike information criterion (AIC) results, five factors (etiology, hemoglobin, creatinine, proteinuria, and UPCR) were selected to establish the predictive nomogram, which is an intuitive visualization of the model (Fig. 2A). According to the constructed model, the risk score of each sample was calculated according to the model coefficients combined with the corresponding value of the five chosen factors. CKD patients were divided into high- (N = 348) and low-risk (N = 348) groups based on their median risk score. Risk score distribution is shown in Fig. 2B. The Kaplan–Meier survival curve of low- and high-risk groups in the training set is shown in Fig. 2C (p < 0.001). The CKD progression status and follow-up time of each individual is shown in Fig. 2D.

### Establishment of a web-based calculator

**Fig. 3:**
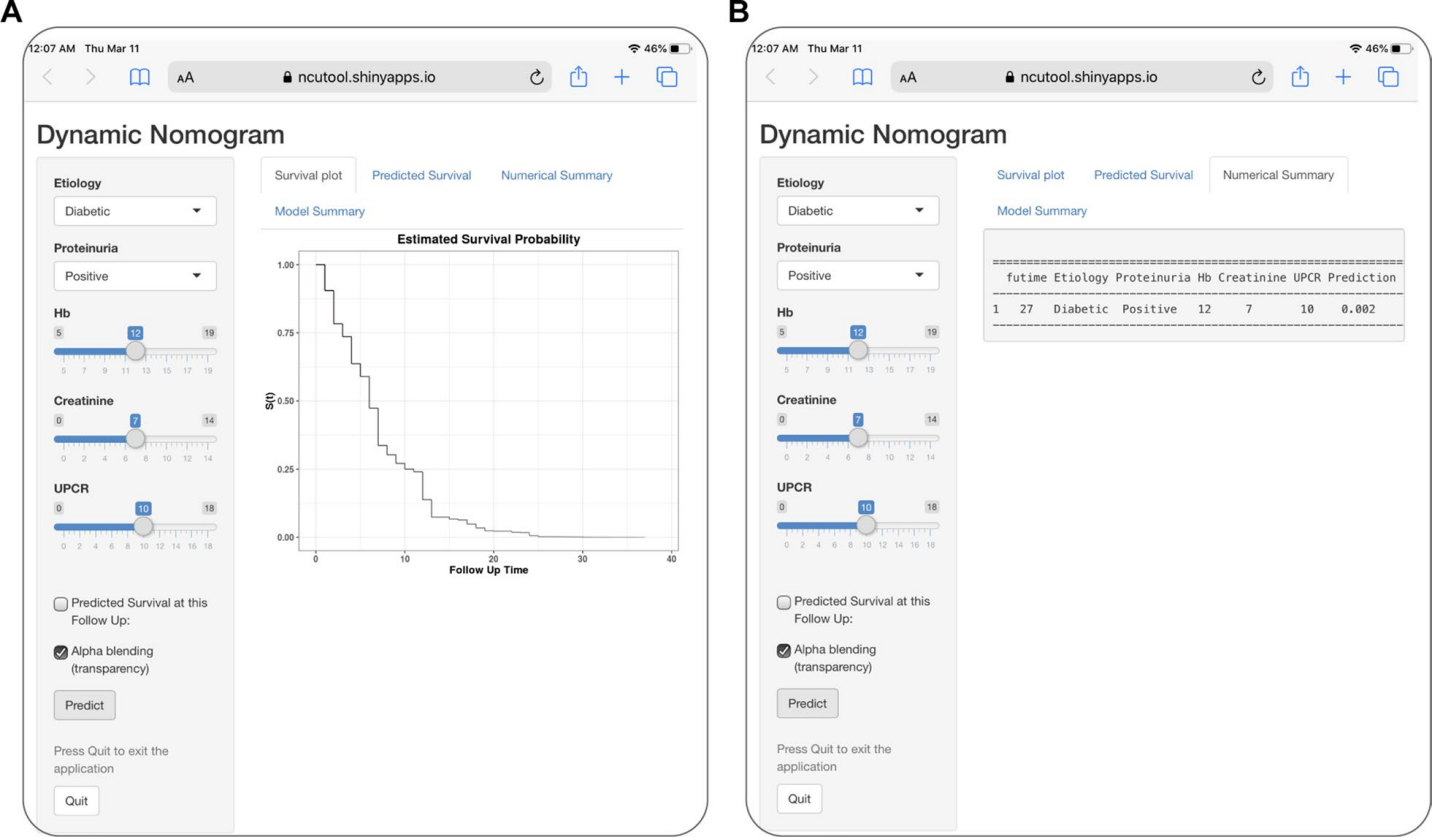
Establishing an easy-to-operate web-based calculator for predicting the progression of chronic kidney disease (https://ncutool.shinyapps.io/CKDprogression/). **A** Web progression-free survival rate calculator. **B** 95% confidence interval of the web progression-free survival rate

For convenient clinical use and visualization of the prognostic model, we developed an easy-to-operate web-based model (https://ncutool.shinyapps.io/CKDprogression/) to predict the progression of CKD based on the established nomogram (Fig. 3). Estimated disease progression probabilities can be obtained by drawing a perpendicular line from the total point axis to the outcome axis.

### Model performance in the training set

**Fig. 4:**
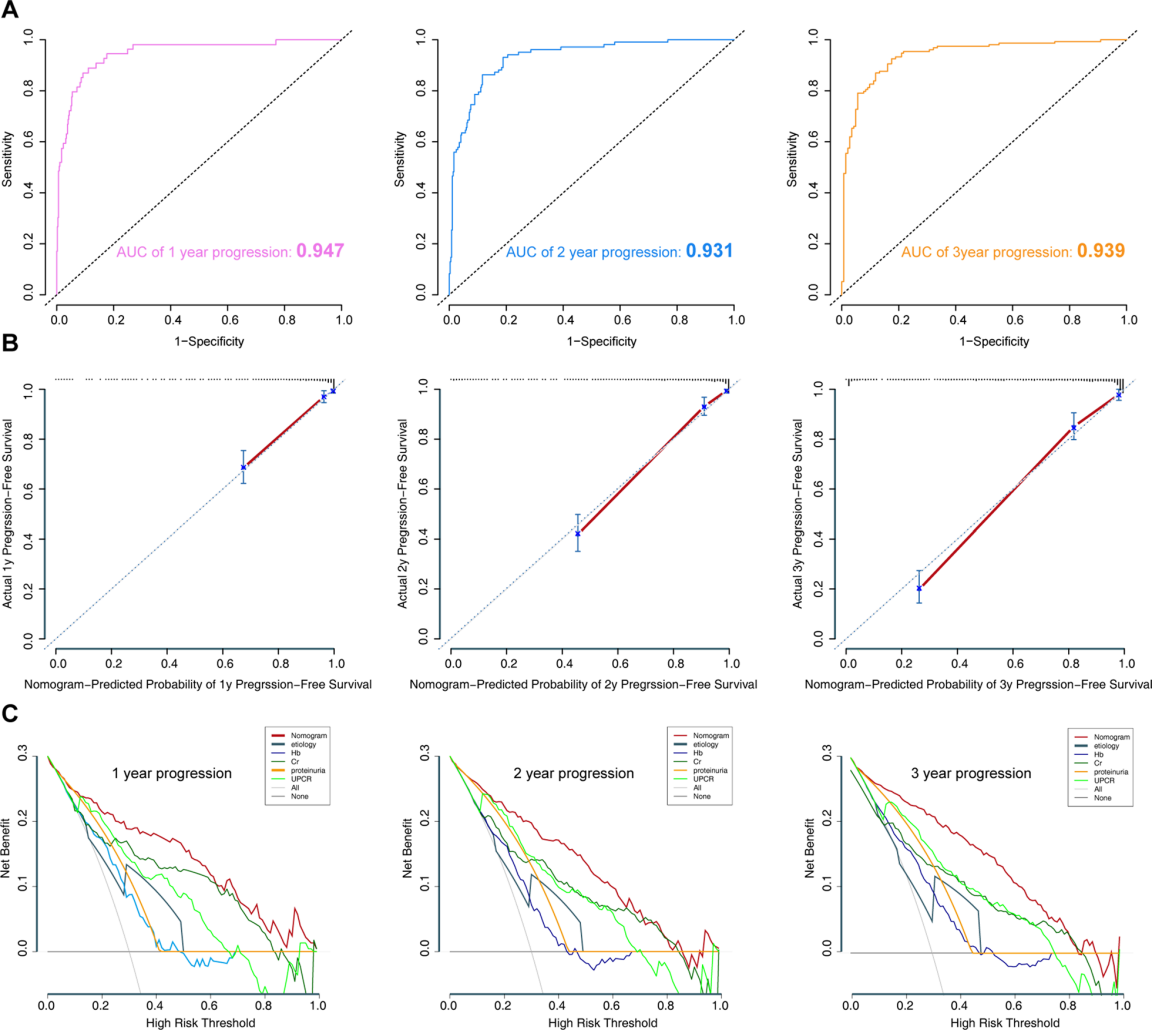
Model discrimination and performance in the training set. **A** Receiver operating characteristic curves for model-based progression-free survival prediction. **B** Calibration plot examining estimation accuracy. **C** Decision curve analysis assessing clinical utility

In the training set, the model was evaluated using time-dependent receiver operating characteristic (ROC) curve analysis over 1-, 2-, and 3-years, along with the concordance index (C-index). The area under the ROC curve (AUC) values for the 1-, 2-, and 3-year survival probabilities were 0.947, 0.931, and 0.939, respectively (Fig. 4A). The C-index for the prediction of progression-free survival was 0.912. The calibration curves of the model showed good probability consistencies between the predicted and observed values (Fig. 4B). These results might confirm that our model was reliable in predicting the prognosis of CKD. Furthermore, a decision curve analysis (DCA) confirmed our expectations, as the analysis revealed that the net benefit in 1-, 2- and 3-year predictions was the highest in the combined nomogram model compared to the single variable (Fig. 4C). Hence, we chose the combined model for clinical use.

### Model performance in the validation set

**Fig. 5:**
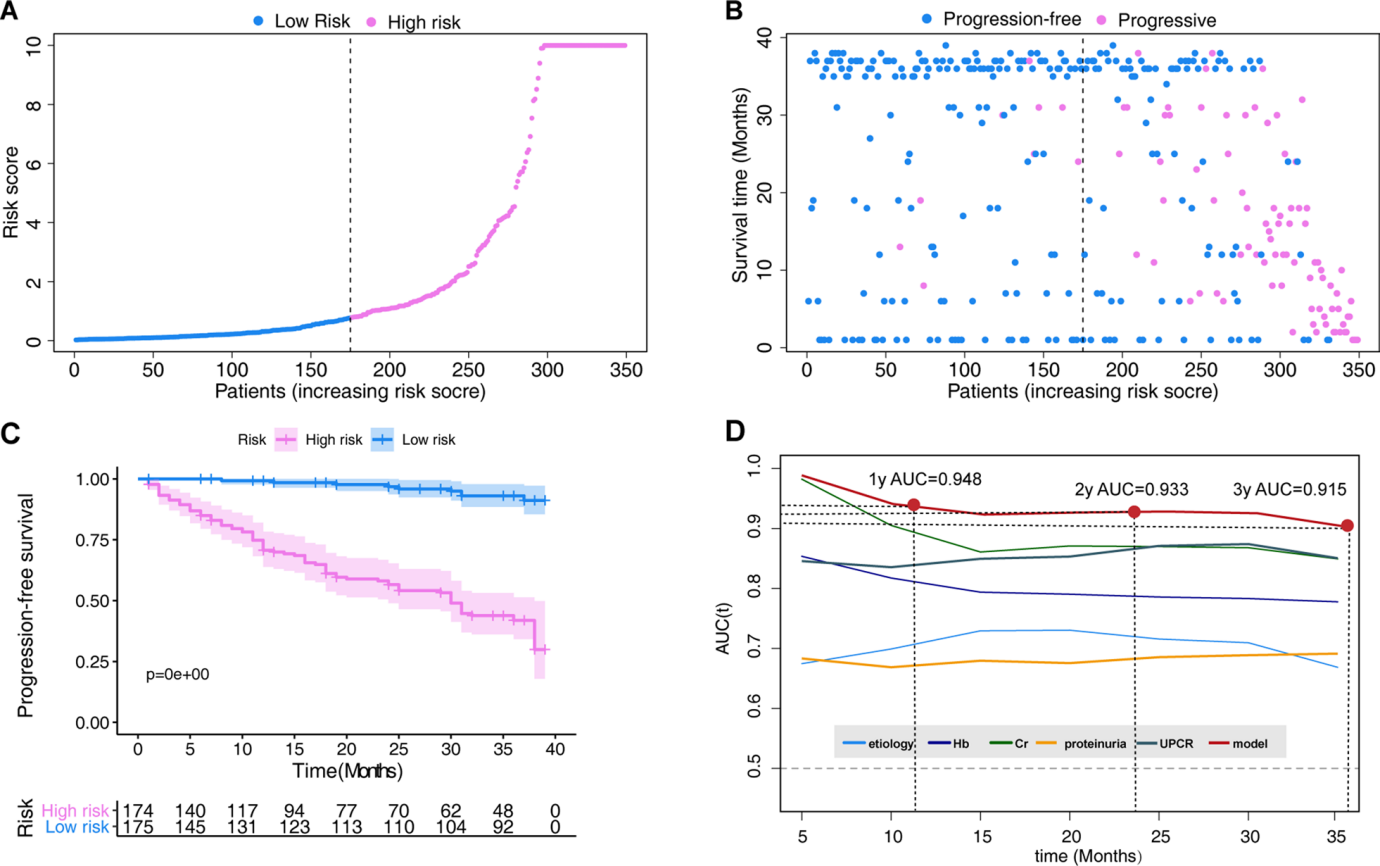
Validation of the nomogram in the validation set. **A** Distribution of the risk scores calculated by the nomogram scoring system. **B** Patient distribution in the low- and high-score groups based on progression status. **C** Progression-free survival curves stratified by the low- and high-score groups. **D** Time-dependent ROC curves for nomogram vs. other single parameters included in the model

In the validation set, CKD patients were divided into high- (N = 174) and low-risk (N = 175) cohorts based on their median risk score. The risk score distribution is shown in Fig. 5A. The CKD progression status and follow-up time of all individuals are shown in Fig. 5B. The Kaplan–Meier survival curve of the low- and high-risk groups is shown in Fig. 5C (p < 0.001). The time-dependent ROC curve analysis validated prediction accuracy of this model over other features (Fig. 5C).

**Fig. 6:**
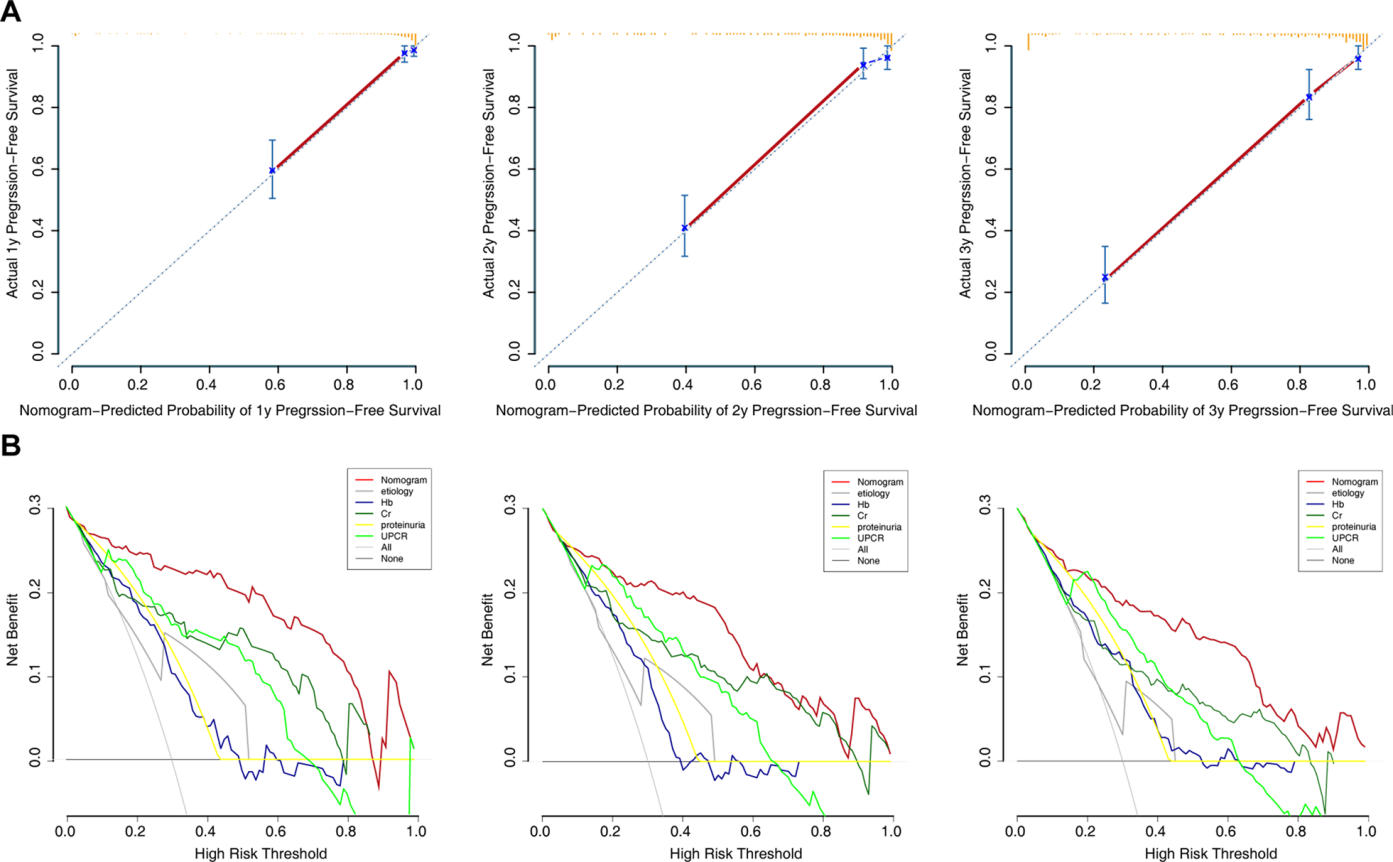
Assessment of the model in the validation set. **A** Calibration plot examining estimation accuracy. **B** Decision curve analysis assessing clinical utility

In addition, we performed calibration plot analysis in the validation set. The calibration curves of the model showed good probability consistencies between the predicted and observed values (Fig. 6A). DCA analysis revealed that the net benefit in the 1-, 2- and 3-year predictions was the highest in the combined nomogram model compared to the single variable (Fig. 6B).

## Discussion

Predicting the CKD outcome in individual patients is beneficial for identifying those who need an aggressive therapeutic regimen. This study showed that etiology, together with proteinuria, serum creatinine, and UPCR, was a better predictor of the risk of progression in patients with CKD when compared to a single indicator. Furthermore, a novel nomogram and corresponding web-based calculator were built as a reference for clinicians to help with clinical decision-making. The risk score identified the highest risk patients accurately, and therefore can identify patients who may benefit most from management by nephrologists without referring the entire population with CKD to them.

The etiology of CKD is multifactorial and diverse. The main causes included diabetes, nephrosclerosis, and glomerulonephritis. In the present study, we found that the highest risk of progression is diabetic kidney disease (DKD). Type 2 diabetes is the most common cause of severe kidney disease worldwide, and DKD is associated with premature death [23]. Although, the fundamental mechanism responsible for the development of DKD to ESRD is poorly understood [24], it is now believed that vessel disease and inflammation are the main pathological mechanisms of CKD [25]. Approximately 40% of diabetic patients develop DKD, and the resultant kidney damage often leads to kidney failure, ultimately requiring dialysis or kidney transplant [26]. Our results suggest that measures should be taken to delay the progression of CKD, especially in cases of DKD.

Proteinuria generally precedes the loss of renal function in kidney disease [27]. For instance, a population-based cohort study in China found that elevated albuminuria was a key predictor of progression to CKD or ESRD and indicated a higher risk of cardiovascular disease and mortality [28]. However, an increasing number of studies have cast doubt on this classic paradigm. In several recent studies, eGFR reduced to 20–39% resulted in normal albuminuria levels [29–31]. In some clinical trials, improvement in proteinuria did not translate to an increased GFR or a reduction in end points such as the need for dialysis or death [32, 33]. The critical role of proteinuria as a single predictor of CKD prognosis requires further study. In the current study, incorporation of several factors including proteinuria might increase model accuracy.

Anemia is a common feature at any stage of CKD, especially in patients with advanced stages of CKD. Anemia in CKD is mainly attributable to the relative decrease in erythropoietin production by the kidneys, absolute or functional iron deficiency, and shortened red blood cell survival. The severity of anemia increases with CKD progression and affects nearly all patients with ESRD [34, 35]. The development of erythropoietic stimulatory agents, such as recombinant human erythropoietin and darbepoetin alpha, has resulted in substantial health benefits for patients with end-stage renal failure, including improved quality of life, reduced blood transfusion requirements, decreased left ventricular mass, diminished sleep disturbance, and enhanced exercise capacity [36, 37]. It is generally believed that low levels of hemoglobin are associated with worse outcomes in patients with CKD [38]. These results are in agreement with the findings of our model.

Previous studies have tried to establish models for progression of CKD to kidney failure [13, 14, 39, 40]. Although the estimations produced by previous models were moderately accurate, the results were somewhat complex because many predictors were involved, each with precise classification levels. We identified five easily accessible and simple demographic and clinical characteristics to include in our novel model, which demonstrated that these traditional factors are important in patients with CKD. Our model showed good calibration and discrimination, and the AUC values generated to predict 1-, 2-, and 3-year progression-free survival in the training set were 0.947, 0.931, and 0.939, respectively. In the validation set, the model revealed excellent calibration and discrimination, and the AUC values generated to predict 1-, 2-, and 3-year progression-free survival were 0.948, 0.933, and 0.915, respectively. These results showed that our model can perfectly predict patient survival in CKD. Moreover, we developed an easy-to-operate calculator that allows the public to freely predict local cases and diagnose the adaptability of the model.

Admittedly, there are some shortcomings in our research. First, the model was developed based on the five variables. However, these factors were unstable throughout the follow-up period, which might have partly influenced the precision of the model. Second, although the performance of the model was good in both the training and validation sets, multicenter clinical application is needed to evaluate the external utility of this model. Third, as the main outcome measure was the progression status of CKD, other outcomes such as survival time should be evaluated in future studies.

In conclusion, we constructed and validated a model incorporating five clinical characteristics (etiology, proteinuria, hemoglobin, creatinine, and UPCR) to predict disease progression in CKD patients. This model could serve as a reliable tool for determining CKD treatment strategies and potential outcomes.

## Data Availability

All the data were obtained from the Dryad Digital Repository (http://www.datadryad.org/).

## Abbreviations

CKD: Chronic kidney disease
AUC: Area under the curve
ESRD: End-stage renal disease
GFR: Glomerular filtration rate
eGFR: Estimated glomerular filtration rate
UPCR: Urinary protein/creatinine ratio
RAAS: Renin–angiotensin–aldosterone-system
AIC: Akaike information criterion
ROC: Receiver operating characteristic
DCA: Decision curve analysis; C-index, concordance index
